# Mitochondriomics of *Clarias* Fishes (Siluriformes: Clariidae) with a New Assembly of *Clarias camerunensis*: Insights into the Genetic Characterization and Diversification

**DOI:** 10.3390/life13020482

**Published:** 2023-02-09

**Authors:** Piyumi S. De Alwis, Shantanu Kundu, Fantong Zealous Gietbong, Muhammad Hilman Fu’adil Amin, Soo-Rin Lee, Hyun-Woo Kim, Ah Ran Kim

**Affiliations:** 1 Department of Marine Biology, Pukyong National University, Busan 48513, Republic of Korea; 2 The Ministry of Livestock, Fisheries and Animal Industries (MINEPIA), Yaoundé 00237, Cameroon; 3 Advance Tropical Biodiversity, Genomics, and Conservation Research Group, Department of Biology, Faculty of Science and Technology, Universitas Airlangga, Surabaya 60115, Indonesia; 4 Research Center for Marine Integrated Bionics Technology, Pukyong National University, Busan 48513, Republic of Korea

**Keywords:** catfish, primer walking, mitogenome, phylogenetics, evolution, diversification

## Abstract

The mitogenome of an endemic catfish *Clarias camerunensis* was determined from the Cameroon water. This circular mitogenome was 16,511 bp in length and comprised 13 protein-coding genes, 2 ribosomal RNAs, 22 transfer RNAs, and a single AT-rich control region. The heavy strand accommodates 28 genes, whereas the light strand is constituted by *ND6* and eight transfer RNA (tRNA) genes. The *C. camerunensis* mitochondrial genome is AT biased (56.89%), as showcased in other *Clarias* species. The comparative analyses revealed that most of the *Clarias* species have 6 overlapping and 11 intergenic spacer regions. Most of the PCGs were initiated and terminated with the ATG start codon and TAA stop codon, respectively. The tRNAs of *C. camerunensis* folded into the distinctive cloverleaf secondary structure, except *trnS1*. The placement of the conserved domains in the control region was similar in all the *Clarias* species with highly variable nucleotides in CSB-I. Both maximum likelihood and Bayesian-based matrilineal phylogenies distinctly separated all *Clarias* species into five clades on the basis of their known distributions (South China, Sundaland, Indochina, India, and Africa). The TimeTree analysis revealed that the two major clades (Indo-Africa and Asia) of *Clarias* species might have diverged during the Paleogene (≈28.66 MYA). Our findings revealed the separation of Indian species (*C. dussumieri*) and African species (*C. camerunensis* and *Clarias gariepinus*) took place during the Paleogene, as well as the South Chinese species (*Clarias fuscus*) and Sundaland species (*Clarias batrachus*) splits from the Indochinese species (*Clarias macrocephalus*) during the Neogene through independent colonization. This pattern of biotic relationships highlights the influence of topography and geological events in determining the evolutionary history of *Clarias* species. The enrichment of mitogenomic data and multiple nuclear loci from their native range or type locality will confirm the true diversification of *Clarias* species in African and Asian countries.

## 1. Introduction

Freshwater fish diversity is rapidly declining around the world due to global warming and severe disruptions of climate change [1]. The catfish family, Clariidae comprises 120 valid species under 16 genera and is distributed in Africa, Syria, and southern and western Asia (Philippines to Java) [2]. The members of Clariidae are remarkable with their general body shape and characterized by suprabranchial organs [3]. This unique organ allows them to acquire oxygen from the environment and survive in hypoxic environments. Identification of walking catfish can sometimes be challenging due to their complex morphology and sexual polymorphism [4,5]. Other than that, the *Clarias* catfishes are popular in aquaculture avenues due to their high commercial value as a nutritious food source and ornamentation [6,7]. They play a significant role in the food web and ecosystems; however, they are confronted with alarming threats due to habitat degradation, over-exploitation, and invasion of alien species [8,9].

Despite the rich species diversity of *Clarias*, a few species (*C. batrachus*, *C. gariepinus*, and *C. macrocephalus*) have been repeatedly studied from different biological and ecological perspectives [10,11]. Further, the North African catfish, *C. gariepinus*, has been used extensively for decades in aquaculture both within and outside its native range in South and Southeast Asian countries (Bangladesh, China, India, Indonesia, Malaysia, Myanmar, North Korea, Philippines, and Thailand), and this great success of aquaculture in tropical countries suggests that this species can be used for cross-breeding with other clariid catfishes [12,13,14,15]. Similarly, the Southeast Asian species, *C. batrachus*, and East Asian species, *C. fuscus,* have extensively spread throughout the world due to aquaculture practices and the aquarium fish trade [16,17,18,19]. Compared to other catfishes, the farming of these *Clarias* species has increased significantly because of their resistance to pathogens and greater adaptability to environmental changes [20,21,22]. Various genetic markers (glutamic acid transfer RNA, cytochrome b, and cytochrome c oxidase subunit 1) were used to elucidate the diversity, evolutionary relationship, population structure, and cryptic diversity of *Clarias* species [23,24,25]. Later on, the complete mitogenomes and whole-genome sequences were analyzed to comprehend the in-depth phylogenetic relationship of *Clarias* species [26,27,28,29,30,31,32,33]. Further, with the flourishment of molecular tools in biodiversity research, the non-invasive environmental DNA (eDNA) was also examined by qPCR assay to reveal the genetic diversity of north African sharptooth catfish, *C. gariepinus* [34]. The mitogenomes of ray-finned fishes (superclass Actinopterygii) are enormously generated throughout the world to elucidate the genetic features and evolutionary relationships [35,36,37]. Until now, a total of 11 mitogenomes of five species (*C. batrachus, C. dussumieri, C. fuscus, C. gariepinus,* and *C. macrocephalus*) have been determined. However, the characterization of the *Clarias* mitogenome, associated gene variation, and phylogeny have been less explored. Further, most previous studies have described specific variations without complete comparison, and thus an overall understanding of the structural variability of the mitogenome of *Clarias* species is not well understood. Hence, the present study was intended to assemble the first complete mitogenome of *Clarias camerunensis* and compare its structure and variations with other *Clarias* mitogenomes. Further, we determined the mitogenome-based phylogeny and time tree to understand the evolutionary pattern and diversification of *C. camerunensis* and other *Clarias* species. Similar studies will help to determine the genetic diversity, phylogeography, and evolutionary scenario, which will help improve the conservation and sustainable usage of *Clarias* species in African and Asian countries. The richness of mitogenomic data will be useful to illuminate the population genetic structure of *Clarias* species across their range distribution, which may be helpful for sustainable conservation of the native species populations and aquaculture practices.

## 2. Materials and Methods

### 2.1. Sampling and Species Identification

A single specimen of *Clarias* fish was collected from the Nyong River (3.760820 N 12.173112 E) in Cameroon and identified as *Clarias camerunensis* on the basis of the previous taxonomic keys [4,5]. The muscle tissue was aseptically excised from the ventral thoracic region after the specimen was euthanized with MS-222 (200 mg/L). The specimen was vouchered in 10% formaldehyde at Fisheries and Animal Industries (MINEPIA), Yaoundé, Cameroon, and the tissue sample was stored at the Department of Marine Biology, Pukyong National University, Busan, South Korea. The experiments were carried out as per the relevant ARRIVE 2.0. (https://arriveguidelines.org) guidelines. No ethics committee or institutional review board approval was required as the muscle of dead fish not killed by the researchers was used. The Institutional Animal Care and Use Committee (IACUC) confirmed that the molecular experiments of biological samples do not belong to any animal ethical issue. The range distribution of *C. camerunensis* (.shp file) was acquired from the IUCN database (https://www.iucnredlist.org/).

### 2.2. DNA Extraction, Mitogenome Sequencing, and Assembly

The AccuPrep^®^ Genomic DNA extraction kit (Bioneer, Republic of Korea) was used to extract the genomic DNA with standard protocol. Both the quality and quantity of the extracted gDNA were checked by using NanoDrop spectrophotometer (Thermo Fisher Scientific D1000). To obtain the complete mitogenome of *C. camerunensis*, the long PCR approach was adopted with primer walking by using different primer pairs (Table S1). Four contigs (*COIII*-*ND6*, *ND6*-CR, CR-*12SrRNA*, CR-*ND1*) were amplified through the conventional method; however, two-step nested PCR was implemented to achieve the other two contigs (*COI*-*COIII*, and *16SrRNA*-*COI*). The PCR was performed by a TaKaRa PCR Thermal Cycler Dice® Gradient (Takara Korea Biomedical Inc. Seoul, Republic of Korea) contained with 1X PCR buffer, 1 U Taq polymerase, 10 pmol primers, 2.5 mM dNTPs, and 1 µL template DNA. The PCR products were further purified by the AccuPrep^®^ PCR/Gel purification kit (Bioneer, Daejeon, Republic of Korea). Later on, each amplicon was amplified with the BigDye^®^ Terminator v3.1 Cycle Sequencing Kit (Applied Biosystems) and sequenced bi-directionally through the ABI PRISM 3730XL DNA analyzer platform available at Macrogen (https://dna.macrogen.com/), Daejeon, Republic of Korea. The noisy parts were trimmed from each chromatogram by using SeqScanner version 1.0 (Applied Biosystems Inc., CA, USA). The mitogenome was assembled by checking the overlying regions alignment through MEGA X [38] and BLAST webserver (https://blast.ncbi.nlm.nih.gov, accessed on 6 February 2023). The boundary of each gene and directions were affirmed through MITOS v806 (http://mitos.bioinf.uni-leipzig.de, accessed on 6 February 2023) and MitoAnnotator (http://mitofish.aori.u-tokyo.ac.jp/annotation/input/, accessed on 6 February 2023) web servers [39,40]. The protein-coding genes (PCGs) were further confirmed through the Open Reading Frame Finder web tool (https://www.ncbi.nlm.nih.gov/orffinder/, accessed on 6 February 2023) after being translated into the putative amino acids of the vertebrate mitochondrial genetic code. The generated mitogenome of *C. camerunensis* was submitted to the GenBank global database.

### 2.3. Genomic Characterization and Comparative Analyses

The spherical view of the *C. camerunensis* mitogenome was designed through MitoAnnotator (http://mitofish.aori.u-tokyo.ac.jp/annotation/input/, accessed on 6 February 2023). A total of 11 mitogenomes of five *Clarias* species (*C. batrachus*, *C. dussumieri*, *C. fuscus*, *C. gariepinus*, and *C. macrocephalus*) were acquired from GenBank for comparative analyses. The overlapping regions and intergenic spacers between the neighbor genes were calculated manually. The nucleotide compositions of PCGs, ribosomal RNA (rRNA), transfer RNA (tRNA), and control region (CR) were calculated using MEGA X. The base composition skews were also calculated as described earlier: AT-skew = [A − T]/[A + T], GC-skew = [G − C]/[G + C] [41]. The initial and termination codons of each PCG were confirmed through MEGA X along with vertebrate mitochondrial genetic code. The rRNA and tRNA gene boundaries were also confirmed through tRNAscan-SE Search Server 2.0 as well as ARWEN 1.2 [42,43]. The structural domains of CR were determined through CLUSTAL X alignments [44], and tandem repeats were investigated by the online Tandem Repeats Finder web tool (https://tandem.bu.edu/trf/trf.html, accessed on 6 February 2023) [45].

### 2.4. Phylogenetic Analyses and Time Tree

To elucidate the matrilineal phylogenetic relationships, a total of 12 mitogenomes (1 generated and 11 databases) of six *Clarias* species were accumulated to build a dataset. The mitogenome of *Heteropneustes fossilis* (family Heteropneustidae) was incorporated into the dataset as an outgroup (Table S2). The iTaxoTools 0.1 tool was used to build a final dataset (11,462 bp) of aligned and concatenated PCGs of all studied mitogenomes [46]. The best fit model ‘GTR+G+I’ was estimated with the lowest BIC value = 88,215.093 in MEGA X. The maximum likelihood (ML) topology was built by MEGA X, and the Bayesian (BA) tree was built by Mr. Bayes 3.1.2 by selecting nst = 6 plus one cold and three hot Metropolis-coupled Markov chain Monte Carlos (MCMCs) and was run for 10,000,000 generations with tree sampling at every 100th generation with 25% of samples rejected as burn-in [47]. The BA tree was illustrated by iTOL v4 webserver (https://itol.embl.de/login.cgi, accessed on 6 February 2023) [48]. The time trees were estimated to obtain chronological information through the MEGAX program with default parameters. The calibration points of median divergent times (*C. camerunensis* vs. *C. gariepinus* and *C. batrachus* vs. *C. fuscus*) were acquired from the global repository of time scale information on the evolution TimeTree of Life resource (TToL5) (http://www.timetree.org/) [49] determined from the earlier studies [25,50,51].

## 3. Results and Discussion

### 3.1. Mitogenomic Structure and Organization

**Figure 1:**
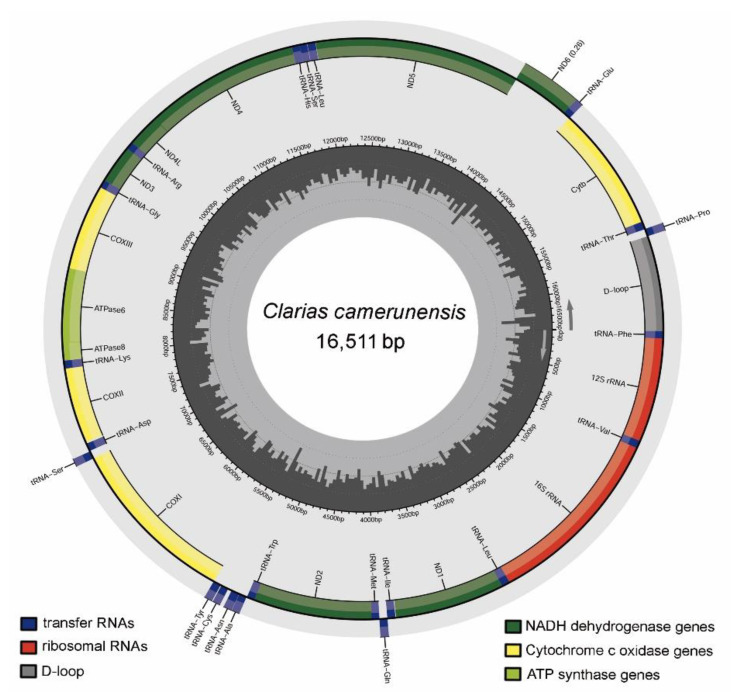
Circular view of the mitochondrial genome of *C. camerunensis*, drawn by the MitoAnnotator online server along with the known range distribution in Angola, Benin, Cameroon, Equatorial Guinea, Gabon, Ghana, Nigeria, Republic of Congo, South Sudan, The Democratic Republic of the Congo, and Togo.

The mitogenome of *C. camerunensis* (16,511 bp) was determined in the present study (GenBank Accession no. OP936082). The generated mitogenome showed length similarity with *C. batrachus* and *C. macrocephalus*. However, considering the highest length, the present mitogenome was longer than C. gariepinus (16,508 bp) and shorter than *C. dussumieri* (16,514 bp) and *C. fuscus* (16,525 bp). The mitogenome of *C. camerunensis* constituted 13 PCGs, 22 tRNAs, 2 rRNAs, and 1 AT-rich CR. The heavy strand accommodated 28 genes (12 PCGs, 2 rRNAs, and 14 tRNAs), while *ND6* and eight tRNAs (*trnQ*, *trnA*, *trnN*, *trnC*, *trnY*, *trnS2*, *trnE*, and *trnP*) were positioned on the light strand (Table 1, Figure 1). The mitogenome of *C. camerunensis* was AT biased (56.89%), with 32.28% A, 14.87% G, 28.24% C, and 24.61% T. Similar AT biasness of the nucleotide composition was also observed in other *Clarias* species ranging from 56.89% (*C. camerunensis*) to 58.63% (*C. dussumieri*). A similar pattern of nucleotide composition and AT biasness was observed in other vertebrate mitogenomes described earlier [52,53]. In the *C. camerunensis* mitogenome, the AT skew and GC skew were 0.135 and −0.310, respectively. The comparative analysis with other *Clarias* mitogenomes showed that the AT skew ranged from 0.119 (*C. fuscus* and *C. macrocephalus*) to 0.223 (*C. dussumieri*), and the GC skew was from −0.310 (*C. camerunensis*) to −0.284 (*C. batrachus*) (Table 2). A total of six overlapping regions with a total length of 25 bp were identified in the *C. camerunensis* mitogenome. The longest overlapping region (10 bp) was observed between ATP synthase 8 (*atp8*) and ATP synthase 6 (*atp6*) genes. Most of the *Clarias* species mitogenomes had six overlapping regions, except for *C. batrachus* (KC572134), with eight overlaps (55 bp), with the longest overlapping region (29 bp) observed between *trnC* and *trnY*. Further, a total of 12 intergenic spacer regions with a total length of 74 bp were observed in *C. camerunensis*, with the longest region (33 bp) between *trnN* and *trnC*. Most of the *Clarias* mitogenomes had 11 intergenic spacer regions, except the lowest in *C. batrachus* (9 with 62 bp) and highest in *C. fuscus* (12 with 78 bp) (Table S3). The genetic variation detected in the *Clarias* mitogenome might be linked with their evolutionary mechanism and energy metabolism, as observed in vertebrates [54]. The study enlightened various structural features of *Clarias* mitogenomes and encoded genes. Such empirical data would be significant for inferring the functions of the mitogenomes and their genes. 

### 3.2. Protein-Coding Genes (PCGs)

In *C. camerunensis*, the length of PCGs was 11,404 bp, which accounted for 69.07% of the total mitogenome. The length of PCGs was lowest in *C. camerunensis* and highest in *C. fuscus* (11,422 bp). In *C. camerunensis* PCGs, the AT skew and GC skew were 0.075 and −0.320, respectively. Most of the PCGs were initiated with ATG start codon excluding *COI* with GTG and *ND5* with ATA. However, the typical termination codon TAA was observed in six PCGs (*ND1*, *COI*, *atp8*, *ND4l*, *ND5*, and *ND6*), except for others with an incomplete stop codon. A similar pattern of initiation codons was observed in all the PCGs; however, variations in stop codons were observed in other *Clarias* mitogenomes (Table S4). In most of the *Clarias* species, the *ND1* gene was terminated by the TAA stop codon, whereas it was TAG in *C. fuscus*. In the *atp6* gene, the incomplete stop codon (TA-) was observed in *C. camerunensis*, *C. batrachus*, and *C. gariepinus* (Table S4). In most of the species, the *COIII* gene was terminated by an incomplete stop codon (T--), while AGG was observed in *C. fuscus*. The mitogenomes of *C. batrachus* and *C. fuscus* showed different stop codons (T-- and TAG) as compared with other species. In the *ND6* gene, three species (*C. camerunensis*, *C. dussumieri*, *C. gariepinus*) revealed the TAA stop codon, while TAG was found in the other three species (*C. batrachus*, *C. fuscus*, *C. macrocephalus*) (Table S4). These incomplete stop codons might be ended with TAA by the adding of a poly A tail over RNA processing [55]. The comparative analysis with other *Clarias* mitogenomes showed that the AT skews ranged from 0.055 (*C. fuscus* and *C. macrocephalus*) to 0.441 (*C. dussumieri*), and the GC skew was from −0.322 (*C. gariepinus*) to -0.297 (*C. batrachus*) (Table 2). The observed genetic variations may lead to the independent selection of PCGs, as observed in other fish species [56,57]. The PCGs encode proteins in the electron transport chains and play crucial roles in oxidative phosphorylation and ATP generation. Therefore, the inclusion of other *Clarias* species mitogenomes can be tested to identify differences in gene expression patterns and energy utilization.

### 3.3. Ribosomal RNA (rRNA) and Transfer RNA (tRNA)

**Figure 2:**
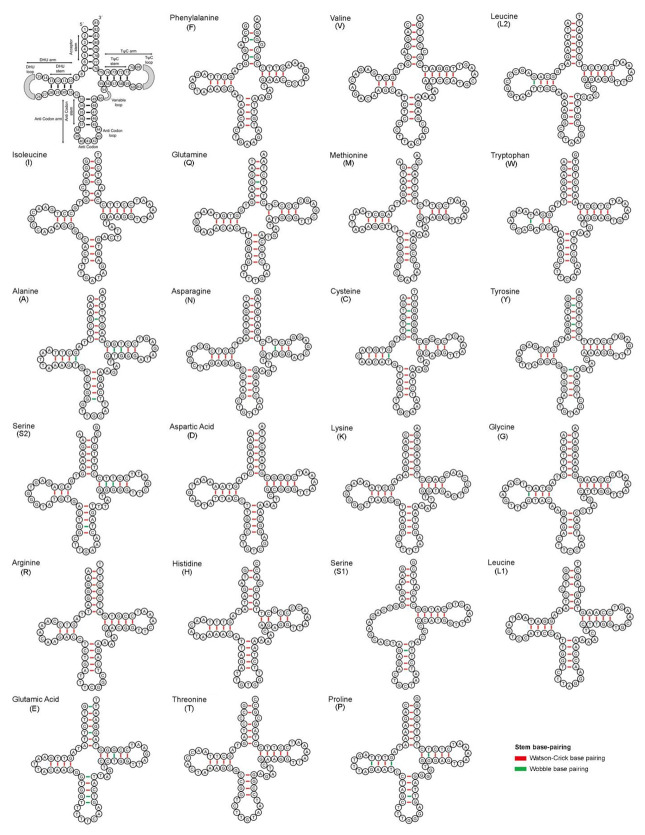
Secondary structures of different tRNA genes of *C. camerunensis* and structural variations in base pairing.

In *C. camerunensis*, the length of ribosomal RNA was 2626 bp (15.90% of the entire mitochondrial genome), encompassing both *12SrRNA* (953 bp) and *16SrRNA* (1673 bp) genes. Compared with other *Clarias* species, the length of ribosomal RNA ranged from 2618 bp (*C. fuscus*) to 2660 bp (*C. batrachus*). The AT richness within the ribosomal RNA ranged from 54.89% (*C. gariepinus*) to 55.5% (*C. dussumieri*). The comparative analysis showed that the AT skew ranged from 0.247 (*C. fuscus*) to 0.325 (*C. dussumieri*), and the GC was skewed from −0.261 (*C. batrachus*) to −0.110 (*C. fuscus*) in the ribosomal RNA (Table 2). The structure and variations in rRNA genes, especially the highly conserved loops, acquire important information on catalytic chemical reaction in protein synthesis [58]. Further, the *C. camerunensis* mitogenome comprises of 22 tRNA genes with a complete length of 1561 bp, which is similar to the *C. macrocephalus* and the highest from other species. The total length of tRNA genes contributed 9.45% of the complete mitogenome of *C. camerunensis* with 56.95% AT composition. The length of tRNA genes ranged from 67 bp (*trnC* and *trnS1*) to 75 bp (*trnL2*). Compared with other *Clarias* species, the AT richness within the transfer RNA ranged from 56.54% (*C. gariepinus*) to 57.2% (*C. macrocephalus*). The range of AT skew was 0.017 (*C. macrocephalus*) to 0.190 (*C. dussumieri*), and GC skew was 0.037 (*C. batrachus*) to 0.054 (*C. macrocephalus*) (Table 2). Most of the tRNAs were folded into the distinctive cloverleaf secondary structures, except *trnS1* (absences of d-arms), as observed in other fishes [37]. These genetic characteristics are important for construction of the secondary structures of RNA and function in diverse living systems [59]. The comparative structural features of tRNA gene revealed 13 tRNA genes (*trnF*, *trnL2*, *trnQ*, *trnW*, *trnA*, *trnN*, *trnC*, *trnY*, *trnS2*, *trnG*, *trnS1*, *trnE*, and *trnP*), which were constituted by both conventional Watson–Crick base (A=T and G≡C) pairing and wobble base pairing (G-T), whereas the other nine tRNA genes only built with the Watson–Crick base pairs (Figure 2).

### 3.4. Control Regions

**Figure 3:**
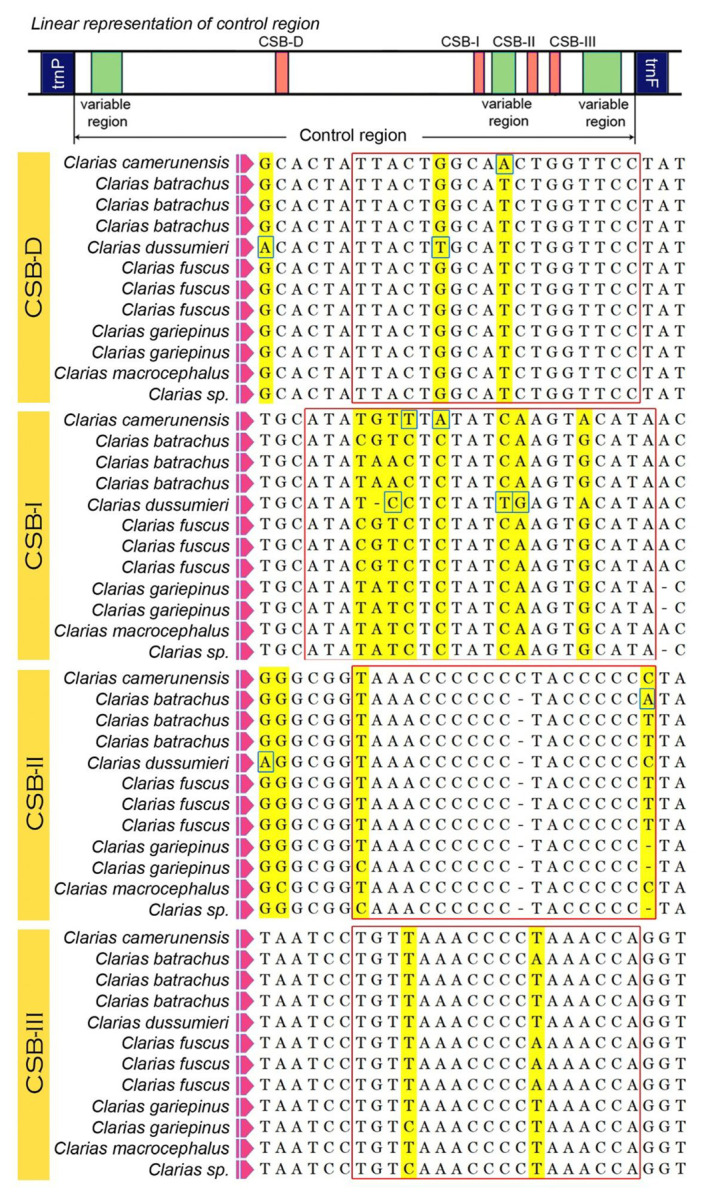
Structural variations in the different conserved domains (marked by red boxes) of the *C. camerunensis* control region and comparison with other *Clarias* species. The variable regions are marked in yellow color, and parsimony informative sites are marked by blue boxes.

The length of *C. camerunensis* CR was 870 bp containing 61.72% AT, which was 5.27% of the complete mitogenome. The size of the CRs in all the *Clarias* species ranged from 863 bp (*C. gariepinus*) to 871 bp (*C. batrachus* and *C. fuscus*). The range of AT skew was −0.019 (*C. fuscus*) to 0.503 (*C. dussumieri*), and GC skew was -0.268 (*C. dussumieri*) to −0.217 (*C. gariepinus*) (Table 2). In *C. camerunensis* CR, two times of the 34 bp tandem repeats were determined in the variable number tandem repeats (VNTRs) region. The size of tandem repeats was similar in other species; however, 2 copies in *C. batrachus*, >1 copy in *C. dussumieri*, and >2 copies in *C. fuscus* were revealed. The similarity of the copy numbers ranged from 88% (*C. camerunensis*) to 97% (*C. fuscus*). No repeats were found in the CR of *C. gariepinus* and *C. macrocephalus*. The placement of the conserved blocks (CSB-D, CSB-I, CSB-II, and CSB-III) was found to be alike in all the studied *Clarias* species, as described in other teleost fishes [37,60]. The length of CSB-I block was the highest (22 bp) compared to other blocks, namely, CSB-II (19 bp), CSB-D (18 bp), and CSB-III (18 bp). The CSB-I block is constituted with high variable sites compared to others (Figure 3). The comparative investigation depicted highly variable nucleotide sites, and parsimony informative nucleotides were spotted in CSB-I. Due to the maternal inheritance, an extremely low rate of recombination, and a fast substitution rate, the mitochondrial markers are commonly used to infer the species level identification in fishes and their genetic diversity. The AT-rich control region can be used to estimate the population structure of any species due to high genetic variability. These variable nucleotides can be used to determine the inter- and intra-specific variations of *Clarias* species. Such conserved domains may play a crucial function in the replication and transcription of the mitochondrial genome, as observed in other species [61].

### 3.5. Matrilineal Phylogeny and Divergence Time

**Figure 4:**
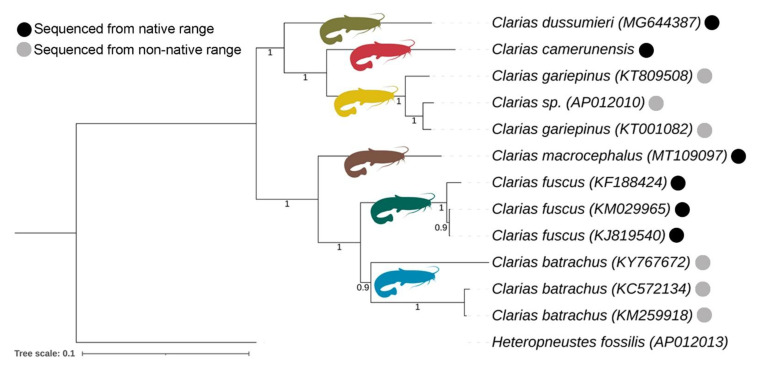
The Bayesian matrilineal phylogeny based on the concatenated sequences of 13 PCGs displays the evolutionary relationship of *C. camerunensis* and other *Clarias* species. The posterior probabilities were overlaid with each node.

The mitogenomic dataset with 13 PCGs exhibited separate clades of all the studied *Clarias* species in the present Bayesian (BA) phylogeny with high posterior probability support. The species (*C. batrachus*, *C. fuscus*, and *C. gariepinus*) with multiple mitogenomes showed monophyletic clustering in the BA topology (Figure 4). The mitogenomes of *C. batrachus* and *C. gariepinus* were not generated from their known native range. However, on the basis of the known range distribution, the present phylogeny showed that the African species (*C. camerunensis* and *C. gariepinus*) are close to each other. One *Clarias* mitogenome (AP012010), identified up to the genus level, cohesively formed a clade with *C. gariepinus*. *Clarias dussumieri* distributed in India showed close association with those in the African clade compared to the East and Southeast Asian species clade. A single mitogenome sequence of *C. batrachus* (KY767672) formed a clade distantly from other two *C. batrachus* mitogenomes, a factor that needs further investigation. Further, three East and Southeast Asian species (*C. batrachus*, *C. fuscus*, and *C. macrocephalus*) also showed sister relationships with each other in BA topology (Figure 4). The present mitogenome-based topologies are congruent with the earlier studies [23,25]. However, the limitation of mitogenomic data is that it actually represents maternally inherited linked genes and therefore provides phylogenetic inferences about only one locus, which can be strengthened by adding nuclear DNA (multiple loci) [62].

**Figure 5:**
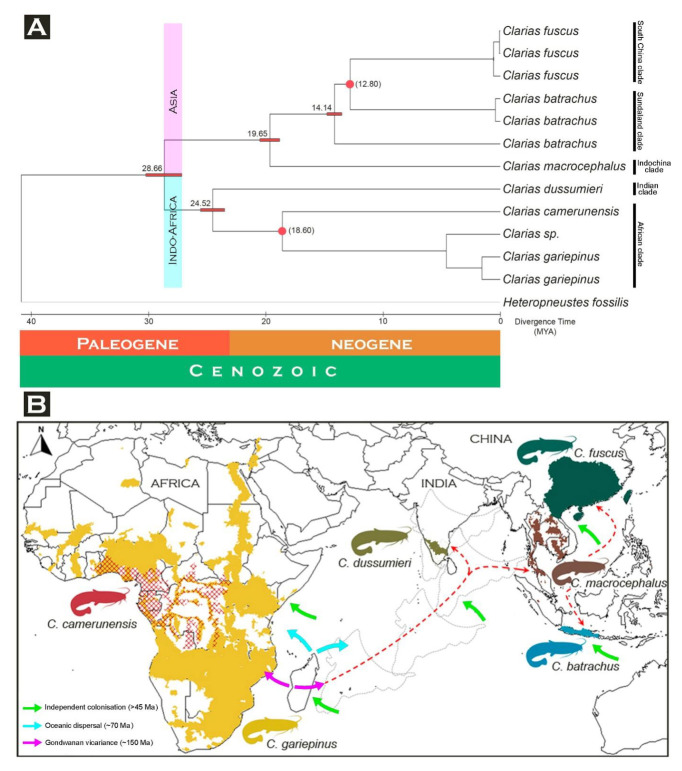
(**A**) The TimeTree elucidates the approximate divergence time of the *Clarias* species. The red dots represent the known calibration points of the targeted species acquired from the TimeTree of Life (http://www.timetree.org/). (**B**) Map showing the possible diversification pattern of *Clarias* species during Cenozoic era.

Molecular dating requires several evolutionary analyses for clarifying the TimeTree and furnishes crucial information for inferring the evolutionary account of any lineages [49,63]. However, the estimation of such divergence time from molecular data is often considered a complex task, which can now be done straightforwardly, even with large sequences and calibration boundaries derived from the previous studies. The recent version of MEGA with the RelTime method is capable of computing a confidence interval of the estimated divergence time. A similar topology was also observed in the maximum-likelihood-based RelTime analysis with high bootstrap support (Figure S1). The mitogenomic data is evidenced to be effective to estimate the time-calibrated phylogeny of catfishes [64]. Considering the most recent diversification of *C. batrachus* and *C. fuscus* (≈12.80 MYR) as well as *C. camerunensis* and *C. gariepinus* (≈18.60 MYR) [25,50,51], the present mitogenomic TimeTree analysis revealed that the two major clades (Indo-Africa and Asia) of *Clarias* species might have diverged during the Paleogene (≈28.66 MYA) (Figure 5A). The present relaxed molecular clock analysis also depicted both *C. batrachus* (distributed in Java, Indonesia) and *C. fuscus* (distributed in China, Hong Kong, Laos, Taiwan, and Vietnam) evolved after *C. macrocephalus*, which is widely distributed in Southeast Asian countries (Cambodia, Laos, Malaysia, Thailand, and Vietnam), and this diversification might have occurred in the Neogene (≈19.65 MYR). The TimeTree also exhibited that *C. dussumieri* distributed in the south-western part of India (Tamil Nadu, Goa, Kerala, Karnataka, Pondicherry) diverged separately compared to the African species (*C. camerunensis* and *C. gariepinus*) during the Paleogene (≈24.52 MYA) (Figure 5A). 

Knowledge of the geographic history of any region is crucial to understanding the shape of its ecological community. It is now widely recognized that the origin and extinction, diversification dynamics, and biogeographic distribution greatly influenced the pattern of species richness. Several studies evidenced that the diversification of organisms occurred during the Cenozoic, and this ancient evolutionary link was established in several ways, with plenty of organisms including fishes [65,66]. The Indo-Eurasian collision and subsequent uplift of the Himalayan–Tibetan Plateau during the Cenozoic era is one of the most imperative geological events in the world [67]. This tectonic movement of the Indian plates separated from the African plate and collision with the Eurasian plate activated biotic exchanges between landmasses [68,69,70,71]. This study discusses the possible biogeographical affinities of *Clarias* fishes by mitogenomic-based phylogeny and TimeTrees. Both the Indian and African *Clarias* showed strong genetic affinity, which due to the connectivity of continents over geologic time led to the biogeographic relationships of the biota being skewed toward the continent with which they were most recently connected [72]. Our findings revealed that the separation of the Indian clade (*C. dussumieri*) from the African clade (*C. camerunensis* and *C. gariepinus*) took place during the Paleogene, which is younger than the initial split of Gondwanan landmasses (i.e., 150 million years) (Figure 5B). Further, the evolutionary split between the Indochina clade (*C. macrocephalus*), Sundaland clade (*C. batrachus*), and South China clade (*C. fuscus*) simultaneously occurred during the Neogene, which is also incongruent with the Oceanic dispersal hypothesis (i.e., 70 million years) (Figure 5B). Hence, our results support that independent colonization played an important role in the evolution of *Clarias* species in both Africa and Asia, which is congruent with the earlier hypothesis [73]. The *Clarias* catfishes evolved independently on all landmasses from a common marine ancestor that has since either become extinct or remains undiscovered. A similar diversification pattern was observed in other fish species across continents [74,75,76]. Such species diversification and colonization are associated with the geoclimatic events and drastic restructuring of climate that occurred throughout the Cenozoic [77]. In the current phylogeny and molecular dating analysis, we understand the limitations of the current dataset involving limited species mitogenomes and believe that further mitogenome development of other species will confirm the true diversification of *Clarias* fishes in African and Asian countries. This pattern of biotic relationships highlights the significance of topography in shaping evolutionary history. Previous studies suggested that the *Clarias* species often showed morphological statis or cryptic diversity in nature. The phenotypic data often fail to discriminate *C. gariepinus* and *C. batrachus*, two distant native ranger species of Africa and Southeast Asia, respectively [78]. The earlier studies have also demonstrated that *C. gariepinus*, historically introduced in Southeast Asia (Thailand), has acquired significant genetic differences from African (Nigeria) *C. gariepinus* [14,79]. Due to unscientific reintroduction of species into non-native ecosystems ignoring native range, human-mediated inter-specific hybridization, or genome manipulation for profitable aquaculture practices, such unparalleled genetic diversity is being revealed in *Clarias* species [13,19,80]. Therefore, the present study recommends analyzing their genetic characteristics from known range distributions and from isolated colonies to know the true genetic structure and evolution of *Clarias* species. Current research endorses the comprehensive genetic screening of *Clarias* species to identify indigenous and non-native populations in the wild as well as scientific breeding to preserve heterozygosity for sustainable aquaculture practices.

## 4. Conclusions

The present paper is dedicated to genetic characterization of the six species of the genus *Clarias*, a member of the family Clariidae, the most important to commercial raising for food source production. We determined the mitogenome sequence of *C. camerunensis* for the first time extending our knowledge about the global evolutionary processes in the genus. The comparative mitogenomic analysis corroborates the systematics and phylogenetic relationship of *C. camerunensis* with other *Clarias* species. The TimeTree also illustrated the independent evolution of *Clarias* species during Cenozoic. Mitogenomic data for *Clarias* species are currently insufficient to construct phylogenies for more than 60 species. We suggest that additional mitogenomes and multiple nuclear loci of this genus will help to gain a comprehensive knowledge of their diversity and evolutionary scenario.

## Figures and Tables

**Table 1:** List of annotated mitochondrial genes of *Clarias camerunensis*.

Genes	Start	End	Strand	Size (bp)	Intergenic Nucleotide	Anti-Codon	Start Codon	Stop Codon
*tRNA-Phe (F)*	1	69	H	69	0	TTC	.	.
*12S rRNA*	70	1022	H	953	0	.	.	.
*tRNA-Val (V)*	1023	1094	H	72	0	GTA	.	.
*16S rRNA*	1095	2767	H	1673	0	.	.	.
*tRNA-Leu (L2)*	2768	2842	H	75	0	TTA	.	.
*ND1*	2843	3817	H	975	5	.	ATG	TAA
*tRNA-Ile (I)*	3823	3894	H	72	−1	ATC	.	.
*tRNA-Gln (Q)*	3894	3964	L	71	−1	CAA	.	.
*tRNA-Met (M)*	3964	4033	H	70	0	ATG	.	.
*ND2*	4034	5078	H	1045	0	.	ATG	T--
*tRNA-Trp (W)*	5079	5149	H	71	3	TGA	.	.
*tRNA-Ala (A)*	5153	5221	L	69	1	GCA	.	.
*tRNA-Asn (N)*	5223	5295	L	73	33	AAC	.	.
*tRNA-Cys (C)*	5329	5395	L	67	7	TGC	.	.
*tRNA-Tyr (Y)*	5403	5472	L	70	1	TAC	.	.
*COI*	5474	7024	H	1551	0	.	GTG	TAA
*tRNA-Ser (S2)*	7025	7095	L	71	4	TCA	.	.
*tRNA-Asp (D)*	7100	7172	H	73	14	GAC	.	.
*COII*	7187	7877	H	691	0	.	ATG	T--
*tRNA-Lys (K)*	7878	7951	H	74	1	AAA	.	.
*ATP8*	7953	8120	H	168	−10	.	ATG	TAA
*ATP6*	8111	8793	H	683	0	.	ATG	TA-
*COIII*	8794	9577	H	784	0	.	ATG	T--
*tRNA-Gly (G)*	9578	9650	H	73	0	GGA	.	.
*ND3*	9651	9999	H	349	0	.	ATG	T--
*tRNA-Arg (R)*	10,000	10,068	H	69	0	CGA	.	.
*ND4L*	10,069	10,365	H	297	−7	.	ATG	TAA
*ND4*	10,359	11,739	H	1381	0	.	ATG	T--
*tRNA-His (H)*	11,740	11,809	H	70	0	CAC	.	.
*tRNA-Ser (S1)*	11,810	11,876	H	67	2	AGC	.	.
*tRNA-Leu (L1)*	11,879	11,951	H	73	2	CTA	.	.
*ND5*	11,954	13,777	H	1824	−4	.	ATA	TAA
*ND6*	13,774	14,292	L	519	0	.	ATG	TAA
*tRNA-Glu (E)*	14,293	14,361	L	69	1	GAA	.	.
*Cyt b*	14,363	15,500	H	1138	0	.	ATG	T--
*tRNA-Thr (T)*	15,501	15,573	H	73	−2	ACA	.	.
*tRNA-Pro (P)*	15,572	15,641	L	70	0	CCA	.	.
Control region	15,642	16,511	H	870	.	.	.	.

**Table 2:** Nucleotide composition of the mitochondrial genome in different *Clarias* species.

Species Name	Size (bp)	A%	T%	G%	C%	A + T%	AT-Skew	GC-Skew
**Complete mitogenome**								
*C. camerunensis*	16,511	32.28	24.61	14.87	28.24	56.89	0.135	−0.310
*C. batrachus*	16,511	32.31	24.97	15.3	27.42	57.28	0.128	−0.284
*C. dussumieri*	16,514	32.52	26.11	14.39	26.97	58.63	0.223	−0.304
*C. fuscus*	16,525	32.17	25.34	14.95	27.54	57.51	0.119	−0.296
*C. gariepinus*	16,508	32.53	24.68	14.84	27.96	57.21	0.137	−0.307
*C. macrocephalus*	16,511	32.2	25.35	14.87	27.58	57.55	0.119	−0.299
**PCGs**								
*C. camerunensis*	11,404	30.66	26.39	14.6	28.35	57.05	0.075	−0.320
*C. batrachus*	11,417	30.61	26.84	14.95	27.6	57.45	0.066	−0.297
*C. dussumieri*	11,408	30.86	28.18	13.99	26.96	59.04	0.441	−0.317
*C. fuscus*	11,422	30.54	27.33	14.56	27.57	57.87	0.055	−0.309
*C. gariepinus*	11,408	30.96	26.51	14.42	28.11	57.47	0.077	−0.322
*C. macrocephalus*	11,408	30.51	27.32	14.54	27.62	57.83	0.055	−0.310
**rRNAs**								
*C. camerunensis*	2626	34.69	20.26	19.46	25.59	54.95	0.263	−0.136
*C. batrachus*	2660	34.51	20.53	20.23	24.74	55.04	0.254	−0.261
*C. dussumieri*	2627	34.64	20.86	19.68	24.82	55.5	0.325	−0.115
*C. fuscus*	2618	34.45	20.82	19.9	24.83	55.27	0.247	−0.110
*C. gariepinus*	2627	34.64	20.25	19.68	25.43	54.89	0.262	−0.127
*C. macrocephalus*	2622	34.48	20.59	19.87	25.06	55.07	0.252	−0.115
**tRNAs**								
*C. camerunensis*	1561	29.34	27.61	22.42	20.63	56.95	0.030	0.042
*C. batrachus*	1560	29.42	27.56	22.31	20.71	56.98	0.033	0.037
*C. dussumieri*	1560	29.17	27.95	22.37	20.51	57.12	0.190	0.043
*C. fuscus*	1560	29.04	27.56	22.56	20.83	56.6	0.026	0.040
*C. gariepinus*	1560	28.85	27.69	22.82	20.64	56.54	0.020	0.050
*C. macrocephalus*	1561	29.08	28.12	22.55	20.24	57.2	0.017	0.054
**CRs**								
*C. camerunensis*	870	30.69	31.03	14.14	24.14	61.72	-0.006	−0.261
*C. batrachus*	871	31.46	31.23	13.89	23.42	62.69	0.004	−0.255
*C. dussumieri*	870	33.22	32.87	12.41	21.49	66.09	0.503	−0.268
*C. fuscus*	871	30.08	31.23	14.24	24.45	61.31	-0.019	−0.264
*C. gariepinus*	863	32.21	30.48	14.6	22.71	62.69	0.028	−0.217
*C. macrocephalus*	869	31.99	30.38	14.15	23.48	62.37	0.026	−0.248

## Data Availability

The genome sequence data that support the findings of this study are openly available in GenBank of NCBI at https://www.ncbi.nlm.nih.gov, accessed on 6 February 2023 under the accession no. OP936082.

## Supplementary Materials

The following supporting information can be downloaded at https://www.mdpi.com/article/10.3390/life13020482/s1. Figure S1: List of designed primer pairs used for the long PCR to assemble *C. camerunensis* mitogenome; Table S2: The mitogenomes of *Clarias* species used in the present analyses; Table S3: Comparison of the intergenic nucleotides of the studied *Clarias* species; Table S4: Start and Stop codons of all 13 PCGS of the studied *Clarias* species; Figure S1: The maximum-likelihood topology with relative times showed the divergence rate of the *Clarias* species. Bootstrap values were superimposed with each node.
