# Supplementary material for: Mitochondriomics of Clarias Fishes (Siluriformes: Clariidae) with a New Assembly of Clarias camerunensis: Insights into the Genetic Characterization and Diversification
Source: Life (Basel). 2023 Feb 9;13(2):482. doi: 10.3390/life13020482 (PMC9960581; doi:10.3390/life13020482)
Supplement: Supplementary file 1 [file life-13-00482-s001.zip › life-2177179-supplementary.pdf]

Supplementary File (s)

Table S1. List of designed primer pairs used for the long PCR to assemble *C. camerunensis* mitogenome.

**Primer Code**	**Sequence (5'-3')**	**Annealing Temp. (°C)**
C1C3_F1	CTAGRTRRGAAGGCCTCGATCC	63.7
C1C3_R1	GTCARTATCADGCAGCKGCTTC	60.6
C1C3_R2	GCAGCKGCTTCRAAKCCRAAGTG	65.4
C3ND6_F1	CGTCTAATGGCCCACCAAGC	64.0
C3ND6_R1	GCTGGDGTDGGDTGTGG	62.7
ND6D1_F1	GAGGAAGYATHATTGCAGG	54.8
ND6D1_R1	GGAACCAGATGCCAGTAATAG	57.2
D112S_F1	GAATCGGCGGMATACCWGTAG	61.3
D112S_R1	GCACCTTCCGGTACACTTAC	60.5
D2ND1_F2	GCATCTGGTTCCTATTTCAGG	58.1
D2ND1_R1	CACTCTATCAAAGTGGYCC	56.5
16SC1_F1	CGTAACAHGGTAAGTGTAYCGG	59.1
16SC1_R1	CCTGTTGGAATTGCRATRATTATTG	56.5
16SC1_R2	GGAATTGCRATRATTATTGTDGC	54.5

Table S2. The mitogenomes of *Clarias* species used in the present analyses.

Sl. No.	Species and Code	Known Distribution	Accession No.	Size (bP)
1	*Clarias camerunensis* Lönnberg, 1895 (CC)	Angola, Benin, Cameroon, Central African Republic, Congo, The Democratic Republic of the Congo, Equatorial Guinea, Gabon, Ghana, Nigeria, South Sudan, and Togo	OP936082	16,511
2	*Clarias batrachus* (Linnaeus, 1758) (CB)	Indonesia (Java)	KY767672	16,496
3			KM259918	16,510
4			KC572134	16,511
5	*Clarias dussumieri* Valenciennes, 1840 (CD)	India (Tamil Nadu, Goa, Kerala, Karnataka, Pondicherry)	MG644387	16,514
6	*Clarias fuscus* (Lacepède, 1803) (CF)	China, Hong Kong, Laos, Taiwan, and Viet Nam	KJ819540	16,507
7			KF188424	16,518
8			KM029965	16,525
9	*Clarias gariepinus* (Burchell, 1822) (CG)	Algeria, Angola, Benin, Botswana, Burkina Faso, Burundi, Cameroon, Central African Republic, Chad, Congo, The Democratic Republic of the Congo, Egypt, Eritrea, Eswatini, Ethiopia, Ghana, Guinea, Israel, Jordan, Kenya, Lebanon, Liberia, Libya, Malawi, Mozambique, Namibia, Niger, Nigeria, Rwanda, Senegal, Somalia, South Africa, South Sudan, Sudan, Syrian Arab Republic, Tanzania, Togo, Turkey, Uganda, Zambia, and Zimbabwe	KT809508	16,505
10			KT001082	16,508
11	*Clarias macrocephalus* Günther, 1864 (CM)	Cambodia, Laos, Malaysia, Thailand, and Viet Nam	MT109097	16,511
12	*Clarias* sp. (CS)	-	AP012010	16,508
13	*Heteropneustes fossilis* (Bloch, 1794)	-	AP012013	16,489

Table S3. Comparison of the intergenic nucleotides of the studied *Clarias* species.

Genes	Species											
	CC	CB1	CB2	CB3	CD	CF1	CF2	CF3	CG1	CG2	CM	CS
tRNA-Phe (F)	0	0	0	0	0	0	0	0	0	0	0	0
12S rRNA	0	0	0	0	0	0	0	0	0	0	0	0
tRNA-Val (V)	0	0	0	0	0	0	0	0	0	0	0	0
16S rRNA	0	0	0	0	0	0	0	0	0	0	0	0
tRNA-Leu (L2)	0	0	0	0	0	0	0	0	0	0	0	0
ND1	5	5	5	5	4	5	5	5	5	5	5	5
tRNA-Ile (I)	-1	-1	-1	-1	-1	-1	-1	-1	-1	-1	-1	-1
tRNA-Gln (Q)	-1	-1	-1	-1	-1	-1	-1	-1	-1	-1	-1	-1
tRNA-Met (M)	0	0	0	0	0	0	0	0	0	0	0	0
ND2	0	0	0	0	0	0	0	0	0	0	0	0
tRNA-Trp (W)	3	3	3	2	3	3	3	3	3	3	3	3
tRNA-Ala (A)	1	1	1	1	1	1	1	1	1	1	1	1
tRNA-Asn (N)	33	32	32	32	31	31	31	31	31	31	32	31
tRNA-Cys (C)	7	10	11	-29	11	10	10	10	11	11	11	11
tRNA-Tyr (Y)	1	1	1	-1	1	1	1	1	1	1	1	1
COI	0	0	0	0	0	0	0	0	0	0	0	0
tRNA-Ser (S2)	4	4	4	4	4	4	4	4	4	4	4	4
tRNA-Asp (D)	14	14	14	14	14	14	14	14	14	14	14	14
COII	0	0	0	0	0	0	0	0	0	0	0	0
tRNA-Lys (K)	1	1	1	1	1	1	1	1	1	1	1	1
ATP8	-10	-10	-10	-10	-10	-10	-10	-10	-10	-10	-10	-10
ATP6	0	0	0	0	0	0	0	0	0	0	0	0
COIII	0	0	0	0	0	0	0	5	0	0	0	0
tRNA-Gly (G)	0	0	0	0	0	0	0	0	0	0	0	0
ND3	0	0	0	0	0	0	0	0	0	0	0	0
tRNA-Arg (R)	0	0	0	0	0	0	0	0	0	0	0	0
ND4L	-7	-7	-7	-7	-7	-7	-7	-7	-7	-7	-7	-7
ND4	0	0	0	0	0	0	0	0	0	0	0	0
tRNA-His (H)	0	0	0	0	0	0	0	0	0	0	0	0
tRNA-Ser (S1)	2	3	2	2	2	2	2	2	2	2	2	2
tRNA-Leu (L1)	2	0	0	0	0	0	0	0	0	0	0	0
ND5	-4	-2	-4	-4	-4	-4	-4	-4	-4	-4	-4	-4
ND6	0	0	0	0	0	0	0	0	0	0	0	0
tRNA-Glu (E)	1	1	1	1	1	1	1	1	1	1	1	1
Cyt b	0	0	0	0	0	0	0	0	0	0	0	0
tRNA-Thr (T)	-2	-2	-2	-2	-2	-2	-2	-2	-2	-2	-2	-2
tRNA-Pro (P)	0	0	0	0	0	0	0	0	0	0	0	0
Control region	.	.	.	.	.	.	.	.	.	.	.	.

Table S4. Start and Stop codons of all 13 PCGS of the studied *Clarias* species.

	Species																							
	CC		CB1		CB2		CB3		CD		CF1		CF2		CF3		CG1		CG2		CM		CS	
**Genes**	Start	Stop	Start	Stop	Start	Stop	Start	Stop	Start	Stop	Start	Stop	Start	Stop	Start	Stop	Start	Stop	Start	Stop	Start	Stop	Start	Stop
ND1	ATG	TAA	ATG	TAA	ATG	TAA	ATG	TAA	ATG	TAA	ATG	TAG	ATG	TAG	ATG	TAG	ATG	TAA	ATG	TAA	ATG	TAA	ATG	TAA
ND2	ATG	T--	ATG	T--	ATG	T--	ATG	T--	ATG	T--	ATG	T--	ATG	T--	ATG	T--	ATG	T--	ATG	T--	ATG	T--	ATG	T--
COI	GTG	TAA	GTG	TAA	GTG	TAA	GTG	TAA	GTG	TAA	GTG	TAA	GTG	TAA	GTG	TAA	GTG	TAA	GTG	TAA	GTG	TAA	GTG	TAA
COII	ATG	T--	ATG	T--	ATG	T--	ATG	T--	ATG	T--	ATG	T--	ATG	T--	ATG	T--	ATG	T--	ATG	T--	ATG	T--	ATG	T--
ATP8	ATG	TAA	ATG	TAA	ATG	TAA	ATG	TAA	ATG	TAA	ATG	TAA	ATG	TAA	ATG	TAA	ATG	TAA	ATG	TAA	ATG	TAA	ATG	TAA
ATP6	ATG	TA-	ATG	TAA	ATG	TAA	ATG	TA-	ATG	TAA	ATG	TAA	ATG	TAA	ATG	TAA	ATG	TAA	ATG	TAA	ATG	TAA	ATG	TA-
COIII	ATG	T--	ATG	T--	ATG	T--	ATG	T--	ATG	T--	ATG	T--	ATG	T--	ATG	AGG	ATG	T--	ATG	T--	ATG	T--	ATG	T--
ND3	ATG	T--	ATG	T--	ATG	T--	ATG	T--	ATG	T--	ATG	T--	ATG	T--	ATG	T--	ATG	T--	ATG	T--	ATG	T--	ATG	T--
ND4L	ATG	TAA	ATG	TAA	ATG	TAA	ATG	TAA	ATG	TAA	ATG	TAA	ATG	TAA	ATG	TAA	ATG	TAA	ATG	TAA	ATG	TAA	ATG	TAA
ND4	ATG	T--	ATG	T--	ATG	T--	ATG	T--	ATG	T--	ATG	T--	ATG	T--	ATG	T--	ATG	T--	ATG	T--	ATG	T--	ATG	T--
ND5	ATA	TAA	ATG	T--	ATG	TAA	ATG	TAA	ATG	TAA	ATG	TAG	ATG	TAG	ATG	TAG	ATG	TAA	ATG	TAA	ATG	TAA	ATG	TAA
ND6	ATG	TAA	ATG	TAG	ATG	TAG	ATG	TAG	ATG	TAA	ATG	TAG	ATG	TAG	ATG	TAG	ATG	TAA	ATG	TAA	ATG	TAG	ATG	TAA
Cyt b	ATG	T--	ATG	T--	ATG	T--	ATG	T--	ATG	T--	ATG	T--	ATG	T--	ATG	T--	ATG	T--	ATG	T--	ATG	T--	ATG	T--

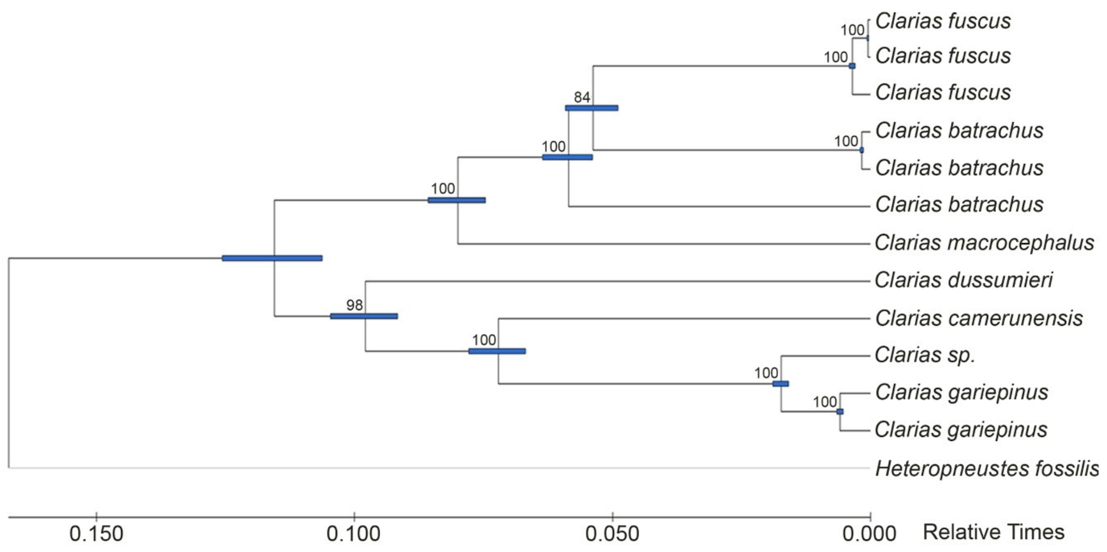

Figure S1. The maximum-likelihood topology with relative times showed the divergence rate of the *Clarias* species. Bootstrap values were superimposed with each node.
